# Menopausal hormone therapy, blood thrombogenicity, and development of white matter hyperintensities in women of the Kronos Early Estrogen Prevention Study

**DOI:** 10.1097/GME.0000000000001465

**Published:** 2020-01-13

**Authors:** Muthuvel Jayachandran, Brian D. Lahr, Kent R. Bailey, Virginia M. Miller, Kejal Kantarci

**Affiliations:** 1 Department of Physiology and Biomedical Engineering, Mayo Clinic, Rochester, MN; 2 Department of Surgery, Mayo Clinic, Rochester, MN; 3 Department of Health Sciences Research, Mayo Clinic, Rochester, MN; 4 Department of Radiology, Mayo Clinic, Rochester, MN.

**Keywords:** 17β-estradiol, Conjugated equine estrogen, Kronos Early Estrogen Prevention Study, Menopause, Postmenopausal, Stroke

## Abstract

**Objective:**

Development of white matter hyperintensities (WMH) in the brain is associated with blood thrombogenicity in recently menopausal women. This study examined the influence of menopausal hormone treatments (MHTs) on this association.

**Methods:**

Measures of blood thrombogenicity were examined in women of the Kronos Early Estrogen Prevention Study (n = 95) who had brain magnetic resonance imaging before and during the 48 months of randomization to transdermal 17β-estradiol (n = 30), oral conjugated equine estrogen (n = 29) both with progesterone for 12 days per month or placebo pills and patch (n = 36). Principal components (PCs) analysis was used to reduce the dimensionality of 14 markers of platelet activation and blood thrombogenicity. The first 5 PCs were assessed for association with treatment and changes in WMH. Within-person slopes were obtained to capture the extent of WMH change for each woman.

**Results:**

WMH increased in all groups over the 48 months (*P* = 0.044). The partial effect of PC_1_, representing an average of six thrombogenicity variables (microvesicles derived from endothelium, leukocytes, and monocytes, and positive for tissue factor and adhesion molecules) on WMH was significant (*P* = 0.003). PC_3_, reflecting a contrast of platelet microaggregates and adenosine triphosphate secretion versus total platelet count, differed across groups (*P* = 0.006) with higher scores in the oral conjugated equine estrogen group. The global association between PCs and WMH increase, however, did not differ significantly by MHT (*P* = 0.207 for interaction between MHT and PC's).

**Conclusion:**

In recently menopausal women, the type of MHT did not significantly influence the association of markers of blood thrombogenicity with development of WMH in the brain.

## Abstract 2

Supplemental Digital Content is available in the text

White matter hyperintensities (WMH) on T2-weighted magnetic resonance imaging (MRI) are associated with ischemic small vessel disease^1,2^ and may precede exhibition of mild cognitive impairment.^3-5^ Conventional cardiovascular risk factors (ie, age, hypertension, smoking, hyperlipidemia) are proposed to be risk factors for development of WMH, especially in elderly persons.^5,6^ Other factors not traditionally considered as cardiovascular risk factors, specifically, thrombogenic microvesicles (MVs), have, however, been implicated in microvascular disease contributing to the formation of WMH.^7-9^ Thrombogenic microvesticles are blood-borne, cell membrane-derived vesicles that carry surface markers of the cell of origin, as well as phospholipids and proteins, associated with coagulation and inflammation.^10^ In recently menopausal women participating in the Kronos Early Estrogen Prevention Study (KEEPS) who were at low risk for cardio- and cerebrovascular disease as defined by a rigorous set of exclusion and inclusion criteria,^11^ WMH increased over the course of the 4 years of the study.^12^ An exploration of nontraditional risk factors that might affect cerebral blood flow and perhaps cerebral microvascular permeability suggested that the thrombogenicity and proinflammatory state of the blood, defined by activated circulating platelets and platelet-derived MVs at the time women enrolled in the study, that is before randomization to treatment, associated with development of WMH over the study course.^12^

KEEPS participants were randomized to either oral conjugated equine estrogen (oCEE), transdermal 17β-estradiol (tE2) both with pulsed progesterone, or placebo pills and patch (PBO) over the 4 years of the study.^11^ These formulations of menopausal hormone treatments (MHTs) were shown to affect platelet secretory products, reactivity, and aggregation.^13-15^ Therefore, the aim of this analysis was to determine whether the type of MHT modified the association of WMH with thrombogenicity of the blood defined by a set of markers of platelet function and reactivity, intravascular cells, cell-derived MVs, endothelial activation, and inflammation.

## METHODS

### Participants

Women enrolled in an ancillary MRI study of the KEEPS (NCT000154180) at Mayo Clinic were eligible for this study. KEEPS was a double-blind, placebo-controlled study to determine the effects of two different hormonal treatments on progression of atherosclerosis defined by increases in carotid intima-medial thickness in recently menopausal women.^11^ In brief, women were between 42 and 59 years old and within 6 months to 3 years past their last menses at the time of enrollment. Women were excluded if they had a coronary artery calcium score of more than 50 Agatston Units, smoked over 10 cigarettes per day, had body mass index more than 35 kg/m^2^, had a history of cardiovascular disease, or had low-density lipoprotein (LDL) cholesterol higher than 190 mg/dL, triglycerides higher than 400 mg/dL, diagnosis of diabetes, uncontrolled hypertension (systolic blood pressure >150 mm Hg and/or diastolic blood pressure >95 mm Hg) or current or recent (6 months) use of cholesterol lowering medications (statins, fibrate, or >500 mg/day niacin). Women were randomized to: oCEE (Premarin, 0.45 mg/day); transdermal tE2 (Climera, 50 μg/day); or PBO pills and patch. Micronized progesterone was given orally (Prometrium; 200 mg/day) for 12 consecutive days each month to both active MHT groups.^11^ The study was approved by the Mayo Clinic Institutional Review Board and all participants gave written informed consent.

### Brain imaging

Women underwent MRI at baseline before randomization, and at 18, 36, and 48 months for measurement of WMH volumes on fluid-attenuated inversion recovery sequences as previously described.^16,17^

### Blood collection and analysis

Women were asked to refrain from aspirin 2 weeks before blood collection. Fasting venous blood was collected into a syringe through a 19 gauge butterfly needle and dispensed into plastic tubes containing anticoagulants needed for each assay and maintained at 33°C until processed within 30 minutes.^18^ Platelet activation using this technique and as measured by surface expression of P-selectin and fibrinogen receptors is less than 5%.^19^ Collections occurred at baseline before randomization to study treatments and at each study time point.^18^ Total cholesterol, LDL cholesterol, and high-density lipoprotein cholesterol, triglycerides, blood glucose, and 17β-estradiol were measured by Kronos Science Laboratories (Phoenix, AZ) and the Mayo Clinic Department of Laboratory Medicine and Pathology (Rochester, MN).

Platelet count was determined by Coulter counter, as previously described.^20^ Expression of activated platelet membrane P-selectin and glycoprotein IIb/IIIa complex binding to PAC-1 antibody (an indirect measure of the expression of membrane fibrinogen receptor) was measured by validated flow cytometry techniques.^18-21^

Total numbers of thrombogenic (phosphatidylserine-positive defined by annexin V binding) MV and other MV staining positive for selected cell-specific markers were measured using fluorophore conjugated recombinant proteins and antibodies by flow cytometry.^18,21,22^

### Statistical analysis

Data reduction methods were used to achieve some degree of parsimony in these analyses so that the complexity could be reasonably supported by the sample size. Analysis of longitudinal WMH data was performed with a two-stage approach to first characterize the time-response profile and then assess differences in profiles across treatment. The goal of the first stage is to adequately summarize the repeat measurements into single WMH responses per person. For this, slope coefficients were computed using least squares regression in fitting each woman's time-response data with a linear equation. To minimize the impact of skewed values, we took the logarithm of WMH, with a constant of 1 added to the raw value, before modeling. One-sample Wilcoxon signed-rank tests for zero slopes were used to test for a significant change in WMH over 48 months separately in each group. The slopes were then assessed for treatment difference using the proportional odds ordinal logistic model, which provides a generalization of the Kruskal-Wallis test for pairwise testing. The results of the two-stage approach were compared with generalized least squares in which all the serial log-transformed data were analyzed jointly in a single model, and the correlation of the repeat measurements taken into account.

For each of the 14 cellular activation markers, repeat measurements on the same person were averaged across visits, and the resulting mean values were converted to normal scores based on their ranks. Scored dimensions of platelet reactivity and MVs were derived using principal component (PC) analysis on the 14 transformed variables. The multiple PCs were carried forward and analyzed for association with three-level treatment in multinomial logistic regression model (an extension of the binary logistic model for >2 unordered outcome categories), and for association with WMH response in a proportional odd ordinal logistic regression model while adjusting for randomized treatment group. *P* values are computed from the likelihood ratio χ^2^ statistic for the model that is due to the individual or multiple variables of interest, with the exception of pairwise treatment comparisons which are based on approximate Wald χ^2^ statistics.

## RESULTS

Baseline characteristics of the 95 participants, a subset of the 118 KEEPS participants at the Mayo Clinic site for whom WMH data were available^12^ did not differ across treatment group assignments except for smoking status (Table 1). WMH increased in all three groups over the 48 months of treatment (*P* < 0.001 each; Table 2). The extent to which WMH increased, as summarized by within-person slopes, differed across treatments (*P* = 0.044), with pairwise comparisons indicating greater increases in oCEE than in PBO (*P* = 0.011). Results from repeated measures modeling (model-predicted WMH at 48 months are shown by treatment group in Supplemental Table 1) were in reasonable agreement with the summary measure analysis, supporting the validity of the simpler two-stage approach, and with differences between oCEE and PBO reported in the original analysis (Tables 1 and 3 of reference 17).

**TABLE 1:** Baseline clinical characteristics of participants by treatment assignment

Variable	N	No. (%) missing	PL (n = 36)	tE2 (n = 30)	oCEE (n = 29)	*P*
Age at randomization, y^*a*^	95	0	52.7 (51.8, 54.3)	53.3 (51.7, 54.3)	53.8 (52.1, 54.8)	0.561
Smoking	80	15 (15.8%)				0.031
Never			25 (73.5%)	13 (56.5%)	16 (69.6%)	
Past			9 (26.5%)	6 (26.1%)	7 (30.4%)	
Current			0 (0.0%)	4 (17.4%)	0 (0.0%)	
Menopausal age, mo (at randomization)^*a*^	95	0	12.7 (10.0, 21.2)	18.5 (14.1, 25.8)	21.4 (10.1, 27.8)	0.108
Body mass index, kg/m^2^^*a*^	95	0	25.7 (24.7, 30.6)	26.8 (22.2, 30.2)	28.4 (25.0, 32.2)	0.330
Waist circumference^*a*^	94	1 (1.1%)	84.8 (76.0, 91.5)	82.5 (72.0, 89.0)	85.0 (77.8, 92.5)	0.564
MSBP^*a*^	95	0	121.8 (114.5, 128.0)	116.5 (110.5, 129.5)	124.0 (112.5, 130.0)	0.538
MDBP^*a*^	95	0	76.0 (70.0, 81.3)	73.8 (66.5, 79.5)	78.5 (71.5, 82.0)	0.282
TC^*a*^	95	0	218.5 (199.5, 232.5)	222.5 (209.0, 248.0)	209.0 (193.0, 237.0)	0.177
HDL^*a*^	95	0	59.5 (50.0, 66.0)	61.0 (53.0, 71.0)	58.0 (49.0, 63.0)	0.371
LDL^*a*^	95	0	136.5 (117.5, 152.3)	150.6 (123.8, 168.6)	133.4 (120.4, 153.4)	0.373
Trig^*a*^	95	0	83.5 (65.0, 107.0)	88.0 (67.0, 118.0)	94.0 (59.0, 117.0)	0.775
hs-CRP^*a*^	92	3 (3.2%)	1.2 (0.5, 2.2)	1.3 (0.4, 2.0)	1.5 (0.8, 3.8)	0.404
Fasting Glucose^*a*^	95	0	91.0 (87.5, 96.5)	94.0 (87.0, 98.0)	89.0 (82.0, 99.0)	0.404

Baseline characteristics of these 95 participants, a subset of the 118 Kronos Early Estrogen Prevention Study (KEEPS) participants at the Mayo Clinic site for whom white matter hyperintensity (WMH) data were available, were published previously._12_ Treatment groups differed with regard to smoking status (*P* = 0.031; four [17.4%] women in the tE2 were smokers at time of baseline, compared with none in the other groups), but were otherwise similar. HDL, high-density lipoprotein; hs-CRP, high sensitivity-C-reactive protein; LDL, low-density lipoprotein; MDBP, mean diastolic blood pressure; MSBP, mean systolic blood pressure; oCEE, oral conjugated equine estrogen; PL, placebo; TC, total cholesterol; tE2, transdermal 17β estradiol; Trig, triglycerides.

^*a*^Median (25th, 75th percentiles); Kruskal-Wallis test.

**TABLE 2:** White matter hyperintensities prior to (baseline) and 18, 36, and 48 months after randomization to placebo or menopausal hormone treatments

White matter hyperintensity volume	N	Placebo pills or patch (n = 36)	Transdermal 17β-estradiol (n = 30)	Oral conjugated equine estrogen (n = 29)	
Baseline	95	1.34 (1.00, 2.02)	1.99 (1.57, 2.85)	2.13 (1.64, 3.66)	
Month 18 visit	92	1.42 (1.01, 2.13)	1.98 (1.64, 2.90)	2.31 (1.67, 3.55)	
Month 36 visit	85	1.52 (1.09, 2.17)	2.13 (1.77, 3.08)	2.59 (1.76, 4.07)	
Month 48 visit	79	1.41 (1.09, 2.35)	2.33 (1.79, 3.38)	2.55 (1.93, 4.63)	
Within-woman slope	95	0.016 (−0.001, 0.028)	0.019 (0.013, 0.035)	0.025 (0.010, 0.051)	P = 0.044^a^

Results reported as median (25th, 75th percentile). For comparison, a generalized least square (GLS) model was fit on log-transformed WMH data, with repeated measurements at 18, 36, and 48 month visits modeled as the response and baseline measurement as a covariate. Results were fairly consistent with the summary measure analysis, based on the interaction between three-level treatment and (linear) time trending toward significance (Wald χ^2^ = 4.8, *P* = 0.089).

^*a*^*P* value is based on the likelihood ratio test from a proportional odds ordinal logistic model, which is a generalization of the Kruskal-Wallis test with pairwise testing fully embedded in the overall model. The significant 2 *df* overall test (likelihood ratio χ^2^ = 6.2, *P* = 0.044) indicates differences in white matter hyperintensity (WMH) rate of increase by treatment group. Pairwise comparisons showed this difference was driven by the oral conjugated equine estrogen versus placebo contrast (Wald χ^2^ = 6.4, *P* = 0.011), with the other two contrasts nonsignificant (Wald χ^2^ = 1.2, *P* = 0.274 for tE2 vs placebo, and Wald χ^2^ = 2.1, *P* = 0.150 for oral conjugated equine estrogen versus transdermal 17β-estradiol).

The average of each of the 14 intravascular cellular activation markers (6 platelet reactivity variables and 8 MV variables) over treatment follow-up is shown by treatment group inTable 3. Because of the multiplicity of variables, and to differences in directional change (increases or decreases compared to placebo) for each of the variables, we used PC analysis to reduce these dimensions to their most important components. The first five PCs were retained as they could explain most (62%) of the variability in the 14 standardized variables (Supplemental Table 2).

**TABLE 3:** Platelet reactivity and blood-borne microvesicles averaged across baseline, 18-, 36-, and 48-month visits

Measurements (averaged over treatment period)	Placebo pills or patch (n = 36)	Transdermal 17β-estradiol (n = 30)	Oral conjugated equine estrogen (n = 29)
Platelet reactivity			
Platelet count (×10^3^/μL)	235.60 (221.70, 260.10)	246.75 (219.40, 287.60)	239.60 (212.60, 264.20)
Platelet microaggregates (% difference)	1.90 (−0.30, 5.04)	2.79 (−0.14, 4.72)	3.58 (0.64, 7.72)
ATP secretion, (attomoles/platelet)	25.78 (22.68, 29.46)	23.96 (20.28, 28.10)	28.90 (23.10, 32.36)
PGE1 sensitivity of ATP secretion (% suppression)	24.92 (15.16, 31.96)	22.05 (16.00, 29.74)	20.30 (14.10, 27.94)
Basal expression of membrane P-selectin (%)	1.61 (1.38, 2.02)	1.40 (1.09, 1.67)	1.72 (1.30, 1.99)
Basal expression of membrane fibrinogen receptor (antibody PAC-1 binding, %)	0.72 (0.64, 0.89)	0.76 (0.61, 0.87)	0.81 (0.64, 0.92)
Microvesicles (MV)/μL plasma			
Phosphatidylserine positive MV	208.84 (170.60, 294.23)	226.94 (141.74, 354.53)	252.54 (178.44, 381.23)
Tissue factor positive MV	22.96 (14.24, 28.62)	23.67 (18.06, 39.35)	17.05 (14.32, 24.97)
Leukocyte (CD45)-derived MV	5.72 (3.48, 10.10)	6.71 (4.83, 8.58)	4.74 (3.73, 6.85)
Monocyte (CD14)-derived MV	16.75 (11.30, 25.42)	16.92 (11.42, 26.96)	17.54 (10.16, 19.07)
Platelet (CD42a)-derived MV	170.95 (132.38, 257.48)	214.58 (135.23, 314.32)	194.14 (141.75, 313.66)
Endothelium (CD62-E)-derived MV	11.17 (8.13, 17.32)	12.96 (8.38, 20.55)	11.96 (9.44, 17.50)
ICAM-1-positive MV	11.16 (7.57, 18.75)	10.45 (5.90, 16.67)	8.98 (5.99, 16.79)
VCAM-1-positive MV	1.18 (0.84, 3.08)	2.02 (0.89, 3.40)	0.91 (0.59, 1.58)

Results reported as median (25^th^, 75^th^ percentile). ATP, adenosine triphosphate; ICAM-1, intracellular adhesion molecule 1; MV, microvesicle; PGE1, prostaglandin E1; VCAM-1, vascular cell adhesion protein 1.

A global test of association with treatment for these five PCs approached significance (*P* = 0.059), with partial tests revealing an overall group difference for PC_3_ (*P* = 0.006). PC_3_ represents a contrast of platelet microaggregates, adenosine triphosphate secretion, basal expression of P-selectin, and fibrinogen receptor complex (PAC-1 binding) versus total platelet count and numbers of leukocyte-derived MV. The composite scores in oCEE were marginally to significantly higher compared to other groups (*P* = 0.003 for oCEE vs tE2, and *P* = 0.063 for oCEE vs PBO; Supplemental Table 3).

Using multivariable regression to test the joint influence of the PCs and treatment on the slope measure for WMH, the global contribution of all five PCs did not reach statistical significance (*P* = 0.104). However, of the individual components PC_1_ reflecting MV positive for expression of tissue factor, intracellular adhesion molecule 1 and vascular cell adhesion protein 1, and MVs derived from leukocytes and monocytes showed the most prominent effect (*P* = 0.003). This finding indicates that, after controlling for treatment, the higher the composite score for PC_1_ the greater the rate of increase in WMH. Also from this model, the association between treatment group and WMH increase persisted after adjustment for PC variables (*P* = 0.009). Based on the global test of interaction on 10 degrees of freedom, there was no evidence (*P* = 0.204) that the overall association between PCs and WMH increase differed by MHT (Table 4).

**TABLE 4:** Modeling rate of change in white matter hyperintensities as a function of principal components and treatment

Model outcome: WMH slope	Model terms	LR χ^2^ test *P* value (*df*)
Model 1: additive model	PC_1-5_	Global, *P *= 0.104 (5 *df*)
	PC_1_	Partial, *P *= 0.003 (1 *df*)
	PC_2_	*P *= 0.953 (1 *df*)
	PC_3_	*P *= 0.961 (1 *df*)
	PC_4_	*P *= 0.634 (1 *df*)
	PC_5_	*P *= 0.853 (1 *df*)
	Treatment	Overall, *P *= 0.006 (2 *df*)
Model 2: nonadditive model	Treatment × PC_1-5_ Interaction	Global, *P *= 0.207 (10 *df*)

*df*, degrees of freedom; PC, principal component; WMH, white matter hyperintensity.

## DISCUSSION

The results of this study support previous observations that blood thrombogenicity and proinflammatory status associate with WMH,^7-9,12^ and extend those observations that this association may be influenced by factors other than the type and dose of menopausal hormones used for the treatment in the Kronos Early Estrogen Prevention Study.

Factors influencing generalized inflammation and, indirectly, blood thrombogenicity, include conventional cardiovascular risk factors such as age, blood pressure, hyperlipidemia, insulin resistance, and life-style choices such as diet, activity, and smoking. In KEEPS, the conventional cardiovascular risk factors such as body mass index, blood pressure, triglycerides, HDL and LDL, glucose, and smoking status, however, did not associate with WMH at 48 months, which is consistent with other studies.^9,12,23,24^ Other potential sources for inflammation in KEEPS participants are unclear. Only 5% of participants were current smokers,^12^ but other behavior factors such as diet and activity were not analyzed, nor were potential sources of commensal or low-grade infectious or inflammatory conditions such as periodontal disease, asthma, or prior histories of hypertensive pregnancy disorders.^25,26^ Each of these conventional and nonconventional risk factors may individually be insufficient to initiate an inflammatory response of the cells within the vascular compartment. Their collective effects of the endothelium, platelets, and monocytes may, however, reach a threshold to alter changes in the macrovasculature (carotid artery intima-media thickness)^18,27^ and cerebral microvasculature affecting development of WMH.

In the present study, the overall association between treatment and the five PCs describing a number of cellular activity measures did not reach statistical significance at the *P* < 0.05 level. The PC_3_ that represented a contrast of platelet reactivity measures, however, differed significantly in the oCEE compared to the tE2 or PBO groups. This result is consistent with previous findings of significant differences in platelet functions between tE2 and oCEE groups.^13,14,15^ Effects of various genetic variants on the responses to treatment might also have masked potential treatment effects as genetic variants associated with metabolism and uptake of estrogen, with innate immunity, and with *APOE* ε4 was observed for MHT effects measured by differences in chronological age for onset of menopause, in carotid artery intima-medial thickness, and deposition of β-amyloid in the brain.^9,28-32^ In spite of these effects, after controlling for treatment, the overall association of the five PCs with increase in WMH reflected the strong positive correlation between PC_1_ score and WMH increase. Taken together these results suggest that both MHT and the composite of the MV measurements explaining PC_1_ show an independent effect on development of WMH.

There are several limitations of this study that should be considered. First, the results may not be applicable to the general population as the KEEPS enrolled recently menopausal women within a relatively narrow age range. In addition, these women were predominantly white, healthy, educated, and most were nonsmokers. The advantage of this homogenous population is that the findings may, however, reflect general physiological processes that are not confounded by manageable cardiovascular risk factors. Second, the influences of the MHT used in KEEPS on development of the WMH may not apply to other doses or formulations of MHT used in other studies. Third, the overall association between PCs and WMH increase did not apparently differ by MHT. However, the relatively small sample in our study may have limited the power to detect such a difference.

## CONCLUSIONS

The findings of the present study are consistent with those of other investigations that implicate thrombogenicity of the blood and inflammation as contributors to development of WMH.^9,12,33^ Activation of blood platelets, endothelium, and monocytes associated with development of WMH are most likely multifactorial including synergistic effects of conventional risk factors such as age, blood pressure, and components of metabolic syndrome. In addition, other potential sources of platelet and cellular activation such as effects of natural menopausal aging processes, adverse pregnancy histories, commensal infections, and comorbid inflammatory conditions and behaviors could have additive effects. Specific mechanisms by which these activated cells and MV affect cerebral microvascular function leading to formation of WMH remain to be determined.

## Supplementary Material

Supplemental Digital Content

## Supplementary Material

Supplemental Digital Content

## Supplementary Material

Supplemental Digital Content

## References

[1] Van Dijk EJ Prins ND Vrooman HA Hofman A Koudstaal PJ Breteler MM Progression of cerebral small vessel disease in relation to risk factors and cognitive consequences: Rotterdam Scan study. *Stroke* 2008; 39:2712–2719. 18635849 10.1161/STROKEAHA.107.513176

[2] DeCarli C Miller BL Swan GE Reed T Wolf PA Carmelli D Cerebrovascular and brain morphologic correlates of mild cognitive impairment in the National Heart, Lung, and Blood Institute Twin Study. *Arch Neurol* 2001; 58:643–647. 11295996 10.1001/archneur.58.4.643

[3] Silbert LC Nelson C Howieson DB Moore MM Kaye JA Impact of white matter hyperintensity volume progression on rate of cognitive and motor decline. *Neurology* 2008; 71:108–113. 18606964 10.1212/01.wnl.0000316799.86917.37 PMC2676966

[4] Kantarci K Weigand SD Przybelski SA Risk of dementia in MCI: combined effect of cerebrovascular disease, volumetric MRI, and 1H MRS. *Neurology* 2009; 72:1519–1525. 19398707 10.1212/WNL.0b013e3181a2e864 PMC2843530

[5] Silbert LC Howieson DB Dodge H Kaye JA Cognitive impairment risk: white matter hyperintensity progression matters. *Neurology* 2009; 73:120–125. 19597134 10.1212/WNL.0b013e3181ad53fd PMC2713187

[6] Debette S Seshadri S Beiser A Midlife vascular risk factor exposure accelerates structural brain aging and cognitive decline. *Neurology* 2011; 77:461–468. 21810696 10.1212/WNL.0b013e318227b227 PMC3146307

[7] Horstman LL Jy W Bidot CJ Potential roles of cell-derived microparticles in ischemic brain disease. *Neurol Res* 2009; 31:799–806. 19723448 10.1179/016164109X12445505689526

[8] Breteler MM Van Swieten JC Bots ML Cerebral white matter lesions, vascular risk factors, and cognitive function in a population-based study: the Rotterdam Study. *Neurology* 1994; 44:1246–1252. 8035924 10.1212/wnl.44.7.1246

[9] Raz N Yang Y Dahle CL Land S Volume of white matter hyperintensities in healthy adults: contribution of age, vascular risk factors, and inflammation-related genetic variants. *Biochim Biophys Acta* 2012; 1822:361–369. 21889590 10.1016/j.bbadis.2011.08.007 PMC3245802

[10] Xiong J Miller VM Li Y Jayachandran M Microvesicles at the crossroads between infection and cardiovascular diseases. *J Cardiovasc Pharmacol* 2012; 59:124–132. 21242813 10.1097/FJC.0b013e31820c6254 PMC3090703

[11] Harman SM Brinton EA Cedars M KEEPS: The Kronos Early Estrogen Prevention Study. *Climacteric* 2005; 8:3–12. 10.1080/13697130500042417 15804727

[12] Raz L Jayachandran M Tosakulwong N Thrombogenic microvesicles and white matter hyperintensities in postmenopausal women. *Neurology* 2013; 80:911–918. 23408873 10.1212/WNL.0b013e3182840c9f PMC3653211

[13] Raz L Hunter LV Dowling NM Differential effects of hormone therapy on serotonin, vascular function and mood in the KEEPS. *Climacteric* 2016; 19:49–59. 26652904 10.3109/13697137.2015.1116504 PMC4834913

[14] Raz L Hunter LW Jayachandran M Heit JA Miller VM Differential effects of oral and transdermal menopausal hormone therapy on prostacyclin and thromboxane in platelets. *Physiol Rep* 2014; 2:e00275. 24760527 10.1002/phy2.275 PMC4002253

[15] Miller VM Lahr BD Bailey KR Heit JA Harman SM Jayachandran M Longitudinal effects of menopausal hormone treatments on platelet characteristics and cell-derived microvesicles. *Platelets* 2016; 27:32–42. 25856160 10.3109/09537104.2015.1023273 PMC4732432

[16] Kantarci K Tosakulwong N Lesnick TG Brain structure and cognition 3 years after the end of an early menopausal hormone therapy trial. *Neurology* 2018; 90:e1404–e1412. 29661902 10.1212/WNL.0000000000005325 PMC5902783

[17] Kantarci K Tosakulwong N Lesnick TG Effects of hormone therapy on brain structure: a randomized controlled trial. *Neurology* 2016; 87:887–896. 27473135 10.1212/WNL.0000000000002970 PMC5035155

[18] Jayachandran M Litwiller RD Lahr BD Alterations in platelet function and cell-derived microvesicles in recently menopausal women: relationship to metabolic syndrome and atherogenic risk. *J Cardiovasc Transl Res* 2011; 4:811–822. 21786187 10.1007/s12265-011-9296-9 PMC3219869

[19] Jayachandran M Litwiller RD Owen WG Heit JA Behrenbeck T Mulvagh SL Characterization of blood borne microparticles as markers of premature coronary calcification in newly menopausal women. *Am J Physiol Heart Circ Physiol* 2008; 295:H931–H938. 18621859 10.1152/ajpheart.00193.2008 PMC2544500

[20] McBane RD 2nd Karnicki K Tahirkheli N Miller RS Owen WG Platelet characteristics associated with coronary artery disease. *J Thromb Haemost* 2003; 1:1296–1303. 12871333 10.1046/j.1538-7836.2003.00183.x

[21] Jayachandran M Miller VM Heit JA Owen WG Methodology for isolation, identification and characterization of microvesicles in peripheral blood. *J Immunol Methods* 2012; 375:207–214. 22075275 10.1016/j.jim.2011.10.012 PMC3253871

[22] Gustafson CM Shepherd AJ Miller VM Jayachandran M Age- and sex-specific differences in blood-borne microvesicles from apparently healthy humans. *Biol Sex Differ* 2015; 6:10. 25964851 10.1186/s13293-015-0028-8 PMC4426551

[23] Kuller LH Margolis KL Gaussoin SA Relationship of hypertension, blood pressure, and blood pressure control with white matter abnormalities in the Women's Health Initiative Memory Study (WHIMS)-MRI trial. *J Clin Hypertens (Greenwich)* 2010; 12:203–212. 20433539 10.1111/j.1751-7176.2009.00234.x PMC2864933

[24] Havlik RJ Foley DJ Sayer B Masaki K White L Launer LJ Variability in midlife systolic blood pressure is related to late-life brain white matter lesions: the Honolulu-Asia Aging study. *Stroke* 2002; 33:26–30. 11779884 10.1161/hs0102.101890

[25] Aukes AM De Groot JC Aarnoudse JG Zeeman GG Brain lesions several years after eclampsia. *Am J Obstet Gynecol* 2009; 200:504 e1-5. 19268882 10.1016/j.ajog.2008.12.033

[26] Raman MR Tosakulwong N Zuk SM Influence of preeclampsia and late-life hypertension on MRI measures of cortical atrophy. *J Hypertens* 2017; 35:2479–2485. 28800041 10.1097/HJH.0000000000001492 PMC5668159

[27] Miller VM Lahr BD Bailey KR Hodis HN Mulvagh SL Jayachandran M Specific cell-derived microvesicles: linking endothelial function to carotid artery intima-media thickness in low cardiovascular risk menopausal women. *Atherosclerosis* 2016; 246:21–28. 26752689 10.1016/j.atherosclerosis.2015.12.030 PMC4764461

[28] Fornage M Debette S Bis JC Genome-wide association studies of cerebral white matter lesion burden: the CHARGE consortium. *Ann Neurol* 2011; 69:928–939. 21681796 10.1002/ana.22403 PMC3122147

[29] Moyer AM De Andrade M Weinshilboum RM Miller VM Influence of SULT1A1 genetic variation on age at menopause, estrogen levels, and response to hormone therapy in recently postmenopausal white women. *Menopause* 2016; 23:863–869. 27300114 10.1097/GME.0000000000000648 PMC4961269

[30] Moyer AM De Andrade M Faubion SS SLCO1B1 genetic variation and hormone therapy in menopausal women. *Menopause* 2018; 25:877–882. 29738412 10.1097/GME.0000000000001109 PMC6092108

[31] Miller VM Jenkins GD Biernacka JM Pharmacogenomics of estrogens on changes in carotid artery intima-medial thickness and coronary arterial calcification: Kronos Early Estrogen Prevention Study. *Physiol Genomics* 2016; 48:33–41. 26508701 10.1152/physiolgenomics.00029.2015 PMC4757027

[32] Kantarci K Lowe VJ Lesnick TG Early postmenopausal transdermal 17beta-estradiol therapy and amyloid-beta deposition. *J Alzheimers Dis* 2016; 53:547–556. 27163830 10.3233/JAD-160258 PMC4955514

[33] Zhang W Huang W Jing F Contribution of blood platelets to vascular pathology in Alzheimer's disease. *J Blood Med* 2013; 4:141–147. 24235853 10.2147/JBM.S45071 PMC3825710

