# Supplementary material for: Menopausal hormone therapy, blood thrombogenicity, and development of white matter hyperintensities in women of the Kronos Early Estrogen Prevention Study
Source: Menopause. 2020 Jan 13;27(3):305–10. doi: 10.1097/GME.0000000000001465 (PMC7050795; doi:10.1097/GME.0000000000001465)
Supplement: Supplemental Digital Content [file menop-27-305-s001.docx]

**Supplemental Table 1.** Generalized Least Squares Model-Predicted 48-mo WMH and Fold-Differences by Treatment

| **Treatment group** | **Predicted WMH at 48m (95% CI)** | **Treatment:**  **PBO fold-difference (95% CI)** |
| --- | --- | --- |
| PBO | 1.96 (1.86 to 2.06) | --- |
| oCEE | 2.07 (1.96 to 2.19) | 1.04 (0.99 to 1.09) |
| tE2 | 2.04 (1.94 to 2.15) | 1.03 (0.98 to 1.08) |
