# Supplementary material for: Menopausal hormone therapy, blood thrombogenicity, and development of white matter hyperintensities in women of the Kronos Early Estrogen Prevention Study
Source: Menopause. 2020 Jan 13;27(3):305–10. doi: 10.1097/GME.0000000000001465 (PMC7050795; doi:10.1097/GME.0000000000001465)
Supplement: Supplemental Digital Content [file menop-27-305-s002.docx]

Supplemental Table 2. Scoring of individual principal components (PC)

| **Measure** | **PC#1** | **PC#2** | **PC#3** | **PC#4** | **PC#5** |
| --- | --- | --- | --- | --- | --- |
| **Platelet Reactivity** |  |  |  |  |  |
| Platelet Count (x10³/μL) |  | **0.27** | *-0.42* | *-0.20* |  |
| Platelet Microaggregates (% difference) |  |  | **0.36** |  | *-0.29* |
| ATP Secretion in Citrate (attomoles/platelet) |  |  | **0.49** |  | *-0.31* |
| PGE1 Sensitivity of ATP secretion (% suppression) |  |  |  | **0.55** | **0.60** |
| Basal Expression of membrane P-Selectin (%) |  |  | **0.34** | **0.35** |  |
| Basal Expression of membrane fibrinogen receptor (PAC-1,%) |  |  | **0.34** | *-0.50* | **0.48** |
| **Microvesicles (MV) / µL plasma** |  |  |  |  |  |
| Phosphatidylserine positive MV |  | **0.64** | **0.20** |  |  |
| Tissue factor positive MV | **0.32** |  |  |  | *-0.29* |
| Leukocyte (CD45) -derived MV | **0.39** |  | *-0.20* | **0.35** |  |
| Monocyte (CD14) -derived MV | **0.47** |  |  |  |  |
| Platelet (CD42a) -derived MV |  | **0.65** |  |  |  |
| Endothelium (CD62-E) -derived MV / | **0.42** |  |  | *-0.21* |  |
| ICAM-1 positive MV | **0.40** |  | **0.25** |  | **0.20** |
| VCAM-1 positive MV | **0.38** |  |  | **0.21** |  |
| **Proportion of Variance Explained by PC** | **19.8%** | **15.2%** | **11.0%** | **8.1%** | **7.7%** |
| **Cumulative Proportion of Variance Explained** | **19.8%** | **35.0%** | **46.0%** | **54.1%** | **61.8%** |

The five principal components (PCs) analysis accounted for 62% of the variability in the original measured components. The first PC, PC_1_, explained 20% of the variance, with substantial component loadings for 6 of the 8 MV variables that ranged between 0.32 and 0.47. The similar contributions of these 6 variables suggest PC_1_ is roughly an average of the corresponding MV measurements. The scoring of PC_2_ is dominated by the remaining 2 MV variables (with loadings of 0.64 and 0.65), whereas the remaining 3 PCs represent contrasts with larger weights for the 6 platelet reactivity variables. Only component loadings with absolute value ≥0.2 are presented in order to emphasize the factors with the strongest contributions to each PC. Italicized values distinguish negative loading.
