# Supplementary material for: Menopausal hormone therapy, blood thrombogenicity, and development of white matter hyperintensities in women of the Kronos Early Estrogen Prevention Study
Source: Menopause. 2020 Jan 13;27(3):305–10. doi: 10.1097/GME.0000000000001465 (PMC7050795; doi:10.1097/GME.0000000000001465)
Supplement: Supplemental Digital Content [file menop-27-305-s003.docx]

Supplemental Table 3. Associations of PC with treatment

|  | **Placebo (n=36)** | **Transdermal 17β estradiol (n=30)** | **Oral conjugated equine estrogen (n=29)** | **LR χ^2^ test**  ***P* value (df)*** |
| --- | --- | --- | --- | --- |
| **PC_1-5_ (61.8%)** |  |  |  | **Global, *P* = 0.059 (10 df)** |
|  |  |  |  |  |
| PC_1_ (19.8%) | 0.36 (-0.86, 1.17) | 0.52 (-0.74, 1.72) | -0.45 (-1.58, 0.49) | Partial, *P* = 0.098 (2 df) |
| PC_2_ (15.2%) | -0.33 (-1.04, 0.59) | 0.21 (-0.86, 1.04) | 0.19 (-0.83, 0.86) | *P* = 0.521 (2 df) |
| PC_3_ (11.0%) | -0.05 (-0.82, 0.73) | -0.88 (-1.33, 0.25) | 0.70 (-0.16, 1.17) | *P* = 0.006 (2 df) |
| PC_4_ (8.1%) | 0.00 (-0.47, 0.76) | -0.14 (-0.86, 0.84) | -0.44 (-0.76, 0.24) | *P* = 0.840 (2 df) |
| PC_5_ (7.7%) | 0.17 (-0.60, 0.72) | 0.09 (-0.89, 0.68) | -0.00 (-0.80, 0.70) | *P* = 0.368 (2 df) |

* Using multi-nomial logistic regression to predict the three-level treatment group from the five principal components, we tested the global null hypothesis of whether any of the five PC is associated with group based on the fitted model’s likelihood ratio χ^2^ test with 10 degrees of freedom. Because this indicated borderline evidence of a group difference for at least one of the PCs, we also report the 2 degrees of freedom (df) partial tests to see which ones are implicated.
